# Antimicrobial resistance profiles and whole-genome sequence analysis of extended-spectrum β-lactamase (ESBL) production in commensal *Escherichia coli* from poultry in Türkiye

**DOI:** 10.1371/journal.pone.0344717

**Published:** 2026-04-08

**Authors:** Seyda Şahin, Büşra Gülay Celil Özaslan, Mahmut Niyazi Moğulkoç, Mehmet Karadağ, Jens Andre Hammerl, Mirjam Grobbel, Cemil Kürekci

**Affiliations:** 1 Department of Food Hygiene and Technology, Faculty of Veterinary Medicine, Sivas Cumhuriyet University, Sivas, Türkiye; 2 Graduate School of Health Sciences, Hatay Mustafa Kemal University, Hatay, Türkiye; 3 Department of Microbiology, Faculty of Veterinary Medicine, Sivas Cumhuriyet University, Sivas, Türkiye; 4 Department of Biostatistics, Faculty of Medicine, Hatay Mustafa Kemal University, Hatay, Türkiye; 5 Department of Biological Safety, German Federal Institute for Risk Assessment, Berlin, Germany; 6 Department of Microbiology, Faculty of Veterinary Medicine, Dokuz Eylül University, İzmir, Türkiye; University of Nicolaus Copernicus in Torun, POLAND

## Abstract

This study examines phenotypic antimicrobial resistance (AMR) and its genetic background in *Escherichia coli* isolated from poultry flocks in Türkiye, with a particular focus on extended spectrum β-lactamase (ESBL)-producing strains. A total of 918 *E. coli* isolates obtained from ceacal samples of chickens (n = 745) and turkeys (n = 173) were subjected to antimicrobial susceptibility testing using the agar disk diffusion method. Overall, high resistance rates were observed to tylosin (99.6%), ampicillin (90.8%), and oxytetracycline (84.0%), while resistance to third-generation cephalosporins (cefotaxime/ceftazidime) was detected in 11.4% of the isolates. Notably, AMR profiles varied significantly between poultry companies, indicating heterogeneous antimicrobial selection pressures within the production sector. ESBL-producing *E. coli* isolates exhibited high levels of multidrug-resistance, particularly to sulfamethoxazole (91.4%) and chloramphenicol (90.5%). Whole-genome sequencing (WGS) of ESBL-producing *E. coli* isolates (n = 87) identified several ESBL-encoding genes, with *bla*_CTX-M-55_ being the most prevalent (51.7%). Plasmid analysis demonstrated frequent associations of *bla*_CTX-M-15_ with IncFIB replicon, while *bla*_CMY-2_ was mainly linked to IncHI2A and IncI1-I plasmid types. In silico typing identified 44 distinct serotypes and 35 sequence types (STs), with O23:H4 and ST1011 being the most detected, highlighting the broad population structure of poultry associated *E. coli*. Virulence-associated genes were widely distributed among ESBL-producing isolates and predominantly related to adhesion, iron acquisition, stress response, and secretion systems. To the best of our knowledge, this study provides the first comprehensive WGS-based analysis of AMR in commensal *E. coli* from poultry in Türkiye, revealing significant public health concerns and the need for enhanced monitoring strategies.

## Introduction

Since the mid-20^th^ century, antimicrobials have been widely used in animal production for therapeutic, prophylactic, and metaphylactic purposes [1,2]. Although antimicrobial utilization has been found to be beneficial for animal health and productivity, it has also accelerated the emergence and spread of antimicrobial resistant bacteria in livestock, thereby contributing to their presence in human populations [3]. The use of antibiotics as growth promoters in food-producing animals has been prohibited in EU countries and in Türkiye since 2006 [4,5] leading to a substantial reduction in veterinary antimicrobial sales. For instance, in the United Kingdom, the sales of veterinary antimicrobial agents decreased from 67.8 mg/PCU (Population Correction Unit) in 2010 to 25.7 mg/PCU in 2022 [5]. Similarly, the Turkish poultry industry has restricted the use of certain antibiotics beyond therapeutic purposes [6]. Several countries have established national surveillance programs to monitor antimicrobial use and resistance in food-producing animals, including DANMAP, MARAN, and NORM-VET [7]. In line with these efforts, Türkiye has also implemented a national antimicrobial resistance (AMR) action plan coordinated by the Ministry of Agriculture and Forestry [8]. Despite these efforts, antimicrobials continue to be widely used in livestock production, and global antimicrobial consumption in animals is projected to increase substaintiall to approximately ~143,481 tonnes by 2040 [2]. This continued use is closely associated with the persistence and dissemination of antibiotic-resistant pathogens. However, in recent years, a decrease in the resistance rates among bacteria from food-producing animals for many substances was notified in European countries, including extended spectrum β-lactamase (ESBL) producing bacteria [9,10].

Commensal bacteria, especially *Escherichia coli*, are commonly used as indicator microorganisms to monitor antibiotic resistance in animals and the environment [11]. Poultry production systems have been identified as important reservoirs of resistant *E. coli*, including resistance to critically important antimicrobials for human medicine [12]. Of particular concern is the increasing resistance to third-generation cephalosporins, which has prompted expanded monitoring of ESBL and AmpC β-lactamases-producing bacteria within a One Health framework [13]. The zoonotic potential of ESBL-producing *E. coli* and the frequent association of CTX-M enzymes with co-resistance to critically important antimicrobials pose a significant threat to human health by limiting treatment options [14,15]. Although recent surveillance reports from Europe indicate generally low resistance levels to third-generation cephalosporins in food-producing animals, ESBL-producing *E. coli* remain a priority concern [7,9].

Although several studies have reported the presence of ESBL-producing *E. coli* in poultry and retail chicken meat samples in Türkiye [16–18], comprehensive data on phenotypic antimicrobial resistance in commensal *E. coli* from poultry, particularly combined with in-depth genomic characterization, remain limited. Moreover, information on the population structure and genetic determinants of ESBL- and AmpC-producing *E. coli* from this source is scarce. It was hypothesized that antimicrobial resistance profiles of commensal *E. coli* differ among poultry farms and production companies in Türkiye, and that ESBL-producing isolates harbor diverse CTX-M subtypes and resistance genes, with potential plasmid-mediated dissemination. Accordingly, the aim of this study was to investigate phenotypic antimicrobial resistance patterns and to characterize the genetic background of ESBL-producing commensal *E. coli* isolated from poultry flocks in Türkiye using whole-genome sequencing (WGS).

## Materials and methods

### Composition of the study collection

Poultry caecal samples were collected in the framework of an ongoing research project on tigecycline-resistant *Enterobacterales* (TUBITAK, Project No: 121N855). Details of sampling are given in Kürekci et al. [19]. Shortly, ceacal samples (n = 940) were taken from healthy animals at slaughter, ten samples per flock from a total of 94 flocks which belonged to chicken companies (A, B and C) and one turkey company (D).

Samples were inoculated onto Eosin Methylene Blue agar (EMB Oxoid, CM0069B, Basingstoke, UK) and cultivated aerobically at 37 ºC for 24 hours. Presumptive *E. coli* colonies (one colony from each sample) were selected based on the appearance of a green metallic sheen and was transferred to Blood Agar Base to obtain a pure strain culture. Species identification of the isolates was performed using a Bruker Biotyper (MALDI-TOF MS, Bruker Daltonics GmbH & Co. KG, Bremen, Germany). Species confirmation was conducted by PCR targeting the *uspA* gene as described by Chen and Griffiths [20].

### Antimicrobial susceptibility testing (AST) by agar disc diffusion

In this study, *E. coli* isolates were further investigated for their susceptibility to 16 antimicrobials/antimicrobial combinations comprising seven antibiotic classes used in human and/or veterinary medicine by using agar disk diffusion assay according to Clinical Laboratory Standard Institute standards [21]. Ciprofloxacin (CIP: 5 µg), enrofloxacin (ENR: 5 µg), florfenicol (FFC: 30 µg), chloramphenicol (CHL: 30 µg), tetracycline (TET: 30 µg), tigecycline (TGC: 15 µg), doxycycline (DO: 30 µg), oxytetracycline (T: 30 µg), trimethoprim-sulfamethoxazole (SXT: 25 µg), gentamycin (CN: 10 µg), amoxicillin/clavulanic acid (AMC: 30 µg), ampicillin (AMP: 10 µg), cefotaxime (FOT: 30 µg), ceftazidime (TAZ: 30 µg), imipenem (IPM: 10 µg), and tylosin (TYL: 15 µg) impregnated discs (Bioanalyse, Ankara, Türkiye) were used in this study. *Escherichia coli* strain ATCC 25922 was used as quality control strain. Isolates were classified as MDR when they exhibit resistance to three or more antimicrobials of distinct classes. According to CLSI [21], susceptibility categories were interpreted; however, intermediate (I) isolates were not evaluated as a distinct category. Instead, they were grouped with susceptible (S) isolates, and antimicrobial susceptibility outcomes were presented dichotomously as susceptible (S) or resistant (R).

### Additional AST by broth microdilution for phenotypic dissection of ESBL-producing *E. coli*

For *E. coli* isolates, that were found to be resistant to TAZ and FOT by agar disc diffusion assay, minimum inhibitory concentrations (MIC) to 15 antimicrobials was further determined by broth microdilution following ISO 20776–1:2019 with commercial plates (Sensititre^TM^ EUVSEC3, Thermo Scientific, UK). The plate-layout included the following antimicrobial agents and ranges: AMP (1–32 mg/L), FOT (0.25–4 mg/L), TAZ (0.25–8 mg/L), meropenem (MERO) (0.03–16 mg/L), azithromycin (AZI) (2–64 mg/L), amikacin (AK) (4–128 mg/L), CIP (0.015–8 mg/L), nalidixic acid (NAL) (4–64 mg/L), CHL (8–64 mg/L), colistin (COL) (1–16 mg/L), CN (0.5–16 mg/L), TET 2–32 mg/L), TGC (0.25–8 mg/L), trimethoprim (TMP) (0.25–16 mg/L) and sulfamethoxazole (SMX) (8–512 mg/L). For quality controls, *E. coli* ATCC 25922 and a carbapenemase-producing *Acinetobacter baumannii* (BfR-AB-00019) were used. MIC results were interpreted according to the European Committee on Antimicrobial Susceptibility Testing (EUCAST) epidemiological cut-off values (http://www.eucast.org), as fixed in Commission Implementing Decision (EU) 2020/1729 (AK > 4 mg/L, AMP > 8 mg/L, FOT > 0.25 mg/L, TAZ > 0.5 mg/L, CHL > 16 mg/L, CIP > 0.06 mg/L, COL > 2 mg/L, CN > 2 mg/L, MERO > 0.125 mg/L, NAL > 8 mg/L, SMX > 64 mg/L, TET > 8 mg/L, TGC > 0.5 mg/L, TMP > 2 mg/L). Isolates were regarded as MDR when they exhibited resistance to three or more antimicrobials from distinct classes [22].

### Whole-genome sequencing and bioinformatics analyses

One ESBL-producing *E. coli* per resistance profile and sample was subjected to short-read WGS on an Illumina NextSeq500 platform (Illumina, San Diego, CA, USA). Genomic DNA (gDNA) for Illumina sequencing libraries were extracted using the PureLink Genomic DNA Mini Kit (Invitrogen/ThermoFisher Scientific) as recommended by the manufacturer’s protocol. Purity and quality parameters of the gDNA were with the Qubit 4.0 Fluorometer according to the standard protocol. Short-read, paired-end sequencing runs were performed in 1x149 cycles on a NextSeq500 benchtop device using the Illumina NextSeq Mid Output Kit v2.5 (300 Cycles) (Illumina, San Diego, CA, USA). After trimming of the raw reads, the sequences were subjected to the AQUAMIS pipeline (https://gitlab.com/bfr_bioinformatics/AQUAMIS) [23]. *de novo* read assembling was performed using the SPAdes (version 3.14.1) of the Bacterial and Viral Bioinformatics Resource Center (BV-BRC) (https://www.bv-brc.org/) [24]. Final annotation of the bacterial genomes was conducted using the automated submission portal (https://submit.ncbi.nlm.nih.gov/) of the National Center for Biotechnology Information (NCBI) under bioproject No PRJNA1168892.

Further in silico-typing purposes (i.e., MLST, cgMLST and serotype prediction) as well as the detection of resistance/virulence genes and plasmids was conducted by using the BakCharak pipeline v3.1.6 (https://gitlab.com/bfr_bioinformatics/BakCharak), including plasmidfinder (db version 2022-07-13) and mlst v2.23.0 with pubmlst database (version 2025-02-27). cgMLST was performed using chewieSnake pipeline (https://gitlab.com/bfr_bioinformatics/chewieSnake). The minimum spanning tree is distance based and was visualized by iTOLv7 using cgMLST profile data (2,513 loci).

### Statistical analysis and visualization

The chi-square test was used to compare the proportions of antimicrobial resistance among companies, with a *p-*value of <0.05 considered statistically significant. The Multiple Antibiotic Resistance Index (MAR) index for ESBL-producing *E. coli* was calculated and interpreted according to Krumperman [25] using the formula: *a*/*b*, where ‘*a*’ represents the number of antibiotics to which an isolate was resistant, and *‘b*’ represents the total number of antibiotics tested. Indices are larger than 0.2 if an isolate originates from a source where antibiotics are used to a great degree and/or in large amounts [26]. Percentage changes in antimicrobial resistance (AMR) rates were calculated using poultry company A as the reference group. The percentage change was determined through the following formula: (CompanyX%−CompanyA%)/CompanyA%×100.

## Results

### Apart from high resistance levels to tylosin, ampicillin and oxytetracycline, the commensal *E. coli* from the four companies exhibit distinct differences in their AMR profiles

**Fig 1:**
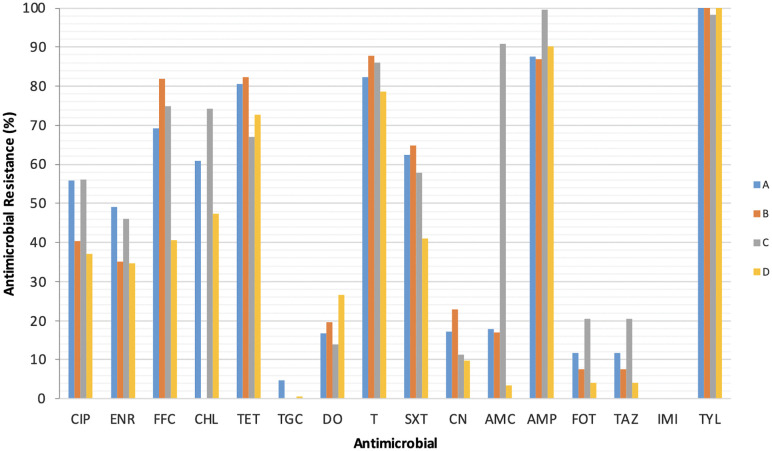
Antimicrobial resistance of *E. coli* strains from avian ceacal swab samples using the agar disk diffusion method (n = 918). A, B, C (chicken companies) and D (turkey company) are representing individual companies. Ciprofloxacin (CIP), enrofloxacin (ENR), florfenicol (FFC), chloramphenicol (CHL), tetracycline (TET), tigecycline (TGC), doxycycline (DO), oxytetracycline (T), trimethoprim-sulfamethoxazole (SXT), gentamycin (CN), amoxicillin/clavulanic acid (AMC), ampicillin (AMP), tylosin (TYL), imipenem (IPM), cefotaxime (FOT) and ceftazidime (TAZ).

A total of 918 *E. coli* (company distribution; A: n = 279, B: n = 236, C: n = 230 and D: n = 173) were obtained from chicken and turkey ceacal samples. AST results from disc diffusion assay is given in Table 1. AMR rates varied significantly among poultry companies (Fig 1), with the highest resistance levels observed for TYL (98.3–100.0%), AMP (86.9–99.6%), T (78.6–87.7%), TET (67.0–80.6%), FFL (40.5–81.8%) and SXT (41.0–64.8%), while DO resistance was moderate in chicken companies (13.9–19.5%). In total, 11.4% and 1.5% of the strains exhibited resistances to FOT/TAZ and TGC, respectively. Resistances against imipenem could not be detected among the isolates. In terms of the antibiotic resistance profiles, significant statistical differences were found for CIP, ENR, FFC, CHL, TET, SXT, CN, AMC, AMP, FOT, and TAZ between companies A, B, C, and D (*p* < 0.001; *p*.001), as summarized in Table 1. Resistance to CN was significantly higher in samples from the three chicken companies compared to those of the turkey company (11.3–22.9% versus 9.8%; *p* < 0.001). Regarding the aminopenicillins, resistance to AMC was low in companies A, B, and D (17.9%, 16.9% and 3.5%, respectively), compared to the very high level in chicken company C (90.9%) (Table 1; Fig 1). Among the 3^rd^ generation cephalosporins, resistance to FOT/TAZ was determined to be 7.6–20.4% in chicken companies (A-C) and 4.0% in the turkey company (D).

**Table 1:** AMR distribution of commensal *E. coli* isolates obtained from swab samples collected from different poultry companies using the agar disk diffusion method (n = 918).

Antimicrobial class	Antimicrobial	Poultry company					
		A	B	C	D	Total	
		n (%)	n (%)	n (%)	n (%)	(%)	p
**Fluoroquinolone**	**Ciprofloxacin**	^a^156 (55.9)	^b^95 (40.3)	^a^129 (56.1)	^b^64 (37.0)	48.4	<.001
	**Enrofloxacin**	^a^137 (49.1)	^b^83 (35.2)	^a^106 (46.1)	^b^60 (34.7)	42.0	.001
**Florfenicol**	**Florfenicol**	^b^193 (69.2)	^a^193 (81.8)	^a,b^172 (74.8)	^c^70 (40.5)	68.4	<.001
	**Chloramphenicol**	^b^170 (60.9)	^a^184 (78.0)	^a^171 (74.3)	^c^82 (47.4)	66.1	<.001
**Tetracycline**	**Tetracycline**	^a,b^225 (80.6)	^a^194 (82.2)	^c^154 (67.0)	^a,c^126 (72.8)	76.1	<.001
	**Tigecycline**	13 (4.7)	0 (0)	0 (0)	1 (0.6)	1.5	na
	**Doxycycline**	^b^47 (16.8)	^a,b^46 (19.5)	^b^32 (13.9)	^a^46 (26.6)	18.6	.010
	**Oxytetracycline**	230 (82.4)	207 (87.7)	198 (86.1)	136 (78.6)	84.0	.060
**Folate biosynthesis pathway inhibitors**	**Trimethoprim/ Sulfamethoxazole**	^a^174 (62.4)	^a^153 (64.8)	^a^133 (57.8)	^b^71 (41.0)	57.7	<.001
**Aminoglycosides**	**Gentamicin**	^a,b^48 (17.2)	^a^54 (22.9)	^b,c^26 (11.3)	^c^17 (9.8)	15.8	.001
**β-Lactams: Penicillin**	**Amoxicillin/Clavulanic acid**	^b^50 (17.9)	^b^40 (16.9)	^a^209 (90.9)	^c^6 (3.5)	33.3	<.001
	**Ampicillin**	^b^244 (87.5)	^b^205 (86.9)	^a^229 (99.6)	^b^156 (90.2)	90.8	<.001
**3**^**rd**^ **generation** **cephalosporins**	**Cefotaxime**	^b^33 (11.8)	^b,c^18 (7.6)	^a^47 (20.4)	^c^7 (4.0)	11.4	<.001
	**Ceftazidime**	^b^33 (11.8)	^b,c^18 (7.6)	^a^47 (20.4)	^c^7 (4.0)	11.4	<.001
**Carbapenem**	**Imipenem**	Nd	Nd	Nd	Nd	Nd	Na
**Macrolide**	**Tylosine**	^a^279 (100)	^a^236 (100)	^b^226 (98.3)	^a^173 (100)	99.6	.007

p value was obtained from Pearson chi square or Fisher exact test. Each subscript letter (a, b and c) denotes a subset of different companies. Nd; not determined. Na; not applicable.

Table 2 displays the percentage changes in antimicrobial resistance rates of poultry companies B, C, and D relative to company A. The most striking finding was the markedly higher resistance in company C to amoxicillin/clavulanic acid (+407.8%) and third-generation cephalosporins (+72.9%). In contrast, company D generally exhibited lower resistance levels across most antimicrobial classes, while complete or near-complete decrease in tigecycline resistance were observed in all companies relative to company A.

**Table 2:** Percentage change in antimicrobial resistance rates relative to poultry company A.

Antimicrobial class	Antimicrobial	B vs A (%)	C vs A (%)	D vs A (%)
**Fluoroquinolone**	**Ciprofloxacin**	−27.9	+0.4	−33.8
	**Enrofloxacin**	−28.3	−6.1	−29.3
**Phenicols**	**Florfenicol**	+18.1	+8.1	−41.5
	**Chloramphenicol**	+28.1	+22.0	−22.1
**Tetracyclines**	**Tetracycline**	+2.0	−16.9	−9.7
	**Tigecycline**	−100.0	−100.0	−87.2
	**Doxycycline**	+16.1	−17.3	+58.3
	**Oxytetracycline**	+6.4	+4.5	−4.6
**Folate pathway inhibitors**	**Trimethoprim/Sulfamethoxazole**	+3.8	−7.4	−34.3
**Aminoglycosides**	**Gentamicin**	−14.5	−23.8	−43.1
**β-lactams**	**Amoxicillin/Clavulanic acid**	−5.6	+407.8	−64.8
	**Ampicillin**	−0.1	+14.1	+5.3
**3**^**rd**^ **generation cephalosporins**	**Cefotaxime**	−35.6	+72.9	−66.4
	**Ceftazidime**	−35.6	+72.9	−66.4
**Macrolide**	**Tylosine**	0.0	−1.7	0.0

### Apart from ESBL-production, high level of MDR was determined for poultry *E. coli*

Of the 918 *E. coli* isolates, a total of 105 *E. coli* displayed phenotypic resistance to 3^rd^ generation cephalosporins in agar disc diffusion assays and are thus assigned as presumptive ESBL-producers and further subjected to broth microdilution testing for MIC determination.

The majority of them revealed MDR patterns, being resistant to antibiotics of more than three classes. As expected, all of these isolates were resistant to AMP (100%), FOT (100%) and TAZ (100%). Further the highest resistance rates were detected for SMX (91.4%; n = 96/105), (CHL (90.5%; n = 95/105), TET (87.6%; n = 92/105), CIP (83.8%; n = 88/105) and NAL (75.2%; n = 79/105). The resistance rate for CN and AZI were (34.3%; n = 36/105) and (21.9%; n = 23/105), respectively. It is also noteworthy that resistance against MERO (1%; n = 1) and COL (1%; n = 1) was detected. However, none of the isolates were found to be resistant to AK or TGC (Table 3). The MAR index of ESBL-producing *E. coli* was found to be SMX 0.91, CHL 0.90, TET 0.87, CIP 0.83, NAL 0.75, TMP 0.67, GEN 0.34, AZI 0.21, MERO 0.01 and COL 0.01.

**Table 3:** MIC distribution of ESBL-producing *E. coli* from poultry caecum samples (n = 105).

Antimicrobial agent	ECOFF value (μg/ml)	Isolates (%) MIC range (μg/ml)																	
		0.015	0.03	0.06	0.125	0.25	0.5	1	2	4	8	16	32	64	128	256	512		% resistant
**Ampicillin**	>8													100					100.0
**Cefotaxime**	>0.25								1.0	4.8	94.3								100.0
**Ceftazidime**	>0.5							1.9	5.7	2.9	33.3	56.2							100.0
**Meropenem**	>0.125		91.4	7.6		1.0													1.0
**Ciprofloxacin**	>0.06	15.2		1.0	1.0	19.0	4.8	6.7	1.9	1.9	33.3	15.2							83.8
**Nalidixic Acid**	>8									21.9	2.9	1.9	1.0	4.8	67.6				75.2
**Azithromycin**	>16								2.9	35.2	28.6	12.4	4.8	7.6	8.6				21.9
**Amikacin**	>8									95.2	4.8								0.0
**Gentamicin**	>2						20.0	41.9	3.8	1.0		2.9	30.5						34.3
**Tigecycline**	>0.5					92.4	7.6												0.0
**Tetracycline**	>8								5.7	6.7			23.8	63.8					87.6
**Chloramphenicol**	>16										9.5	2.9	1.0	8.6	78.1				90.5
**Colistin**	>2							97.1	1.9	1.0									1.0
**Trimethoprim**	>2					26.7	6.7						67.6						67.6
**Sulfamethoxazole**	>64										3.8	4.8						91.4	91.4

MIC values have been interpreted using EUCAST Epidemiological cut-off (ECOFF) values, which are displayed as vertical black lines. Black and white background indicates the concentrations tested with the EUVSEC3 plate-layout.

### CTX-M is the most prevalent enzyme conferring ESBL status within the genetically highly diverse *E. coli* collection

In this study, 99 ESBL-producing *E. coli* strains were selected for WGS. Based on the SNP analysis, only one clone per flock was selected for further in-depth analyses, leaving 87 ESBL-conferring *E. coli* genomes. WGS provided detailed insights about individual genetic determinants responsible for the observed phenotypic resistances and indicate the broad diversity of the occurring *E. coli* populations in chicken and turkey. Among the genomes, five different *bla*_CTX-M_ genes were identified. The most abundant are *bla*_CTX-M-55_ accounting for 51.7% (n = 45/87) of the isolates, followed by *bla*_CTX-M-15_ (18.4%; n = 16/87), *bla*_CTX-M-1_ (3.4%; n = 3/87), *bla*_CTX-M-27_ (1.1%; n = 1/87), and *bla*_CTX-M-8_ (1.1%, n = 1/87) (Table 4). Additionally, AmpC β-lactamase genes *bla*_CMY-2_ 23.0% (n = 20/87) and *bla*_DHA-1_ were found in 2.3% (n = 2/87) of the isolates. In addition, also non-ESBL *bla*_TEM_ gene variants like *bla*_TEM-1A,_ *bla*_TEM-1B,_ *bla*_TEM-1C_ and *bla*_TEM-176_ were found in 62.1% (n = 54/87) of the isolates, while *bla*_OXA-1_ and *bla*_OXA-4_ occur in 1.1% (n = 1/87) and 10.7% (n = 3/87) of the isolates, respectively.

**Table 4:** Distribution of β-lactamase genes among ESBL-producing *E. coli* from four integrated poultry companies (n = 87).

		No. of isolates carrying each gene (%)				
Enzyme	β-Lactamase Gene	A (n = 28)	B (n = 15)	C (n = 38)	D (n = 6)	Total (n = 87)
**Narrow spectrum** **β-Lactamase**	*bla*_TEM-1A_	0 (0.0)	0 (0.0)	0 (0.0)	1 (16.7)	**1 (1.1)**
	*bla*_TEM-1B_	16 (57.1)	12 (80.0)	20 (52.6)	2 (33.3)	**50 (57.5)**
	*bla*_TEM-1C_	0 (0.0)	0 (0.0)	1 (2.6)	0 (0.0)	**1 (1.1)**
	*bla*_TEM-176_	1 (3.6)	0 (0.0)	0 (0.0)	0 (0.0)	**1 (1.1)**
	*bla*_OXA-1_	0 (0.0)	0 (0.0)	0 (0.0)	1 (16.7)	**1 (1.1)**
	*bla*_OXA-4_	3 (10.7)	0 (0.0)	0 (0.0)	0 (0.0)	**3 (3.4)**
**Extended spectrum** **β-Lactamase**	*bla*_CTX-M-1_	2 (7.1)	1 (6.7)	0 (0.0)	0 (0.0)	**3 (3.4)**
	*bla*_CTX-M-8_	1 (3.6)	0 (0.0)	0 (0.0)	0 (0.0)	**1 (1.1)**
	*bla*_CTX-M-15_	3 (10.7)	5 (33.3)	5 (13.2)	3 (50.0)	**16 (18.4)**
	*bla*_CTX-M-27_	0 (0.0)	1 (6.7)	0 (0.0)	0 (0.0)	**1 (1.1)**
	*bla*_CTX-M-55_	9 (32.1)	5 (33.3)	28 (73.7)	3 (50.0)	**45 (51.7)**
**AmpC β-Lactamase**	*bla*_CMY-2_	11 (39.3)	3 (20.0)	5 (13.2)	1 (16.7)	**20 (23.0)**
	*bla*_DHA-1_	2 (7.1)	0 (0.0)	0 (0.0)	0 (0.0)	**2 (2.3)**

A, B, C = Chicken company D = Turkey company. Values in parentheses represent percentage proportions of isolates carrying the indicated genes.

Overall, 44 different serotypes were identified in-silico among the 87 ESBL-producing *E. coli*. Both O and H antigens were predicted for 94.3% (n = 82) of the strains, however the O antigens of five genomes (5.68%) were not typeable. The most common detected serotypes were O23:H4 (6.8%, n = 6/87), followed by O83:H42 (5.7%, n = 5/87) and O9:H12, O18:H49, O100:H30, O102:H9, O136:H26, and O160:H4 (3.4%, n = 3/87). Other commonly recorded serotypes are given in S1 Table.

### Commensal *E. coli* from poultry exhibit a broad set of AMR determinants

Different proportions of strains harbored genes encoding for resistance to tetracycline’s [*tet(A)* (n = 76) and *tet(B)* (n = 8)], quinolone [*qnrS1* (n = 6); *qnrB4* (n = 2); *qnrB19* (n = 8) and *aac(6‘)-Ib-cr* (n = 1)], folate pathway inhibitors [(*dfrA1* (n = 53), *dfrA5* (n = 5), *dfrA12* (n = 10), *dfrA14* (n = 15), *dfrA17* (n = 23) and *dfrA36* (n = 1)], sulfonamide [*sul1* (n = 13), *sul2* (n = 54) and *sul3* (n = 37)], macrolides [*mph(A)* (n = 18), *mph(B)* (n = 1), *erm(42)* (n = 1) and *erm(B)* (n = 1)], lincosamides [*lnu(F)* (n = 25) and *lnu(G)* (n = 1)], phenicols [*catA1* (n = 8), *catB2* (n = 4), *cmlA1* (n = 33), *catB3* (n = 4) and *floR* (n = 73)], aminoglycosides [*aac(3)-IId* (n = 23), *aac(3)-IIe* (n = 6), *aadA1* (n = 43), *aadA2* (n = 36), *aadA5* (n = 21), *aadA22* (n = 1), *ant(2’‘)-Ia* (n = 4), *aph(3’)-Ia* (n = 38), *aph(3’‘)-Ib* (n = 54), *aph(3’)-XV* (n = 3), *aph(4)-Ia* (n = 1), *aph(6)-Id* (n = 54) and *aac(6‘)-Ib-cr* (n = 1)]. Furthermore, we detected the *mcr*-1 gene in one colistin-resistant strain from a chicken caecum sample (S1 Table).

### A high genetic diversity was determined in the representative commensal *E. coli* study strain collection

The majority of the 87 ESBL-producing *E. coli* were assigned to phylogenetic groups B1 (27.6%, n = 24), A (25.3%, n = 22) and D (24.1%, n = 21). Phylogenetic group F (13.8%, n = 12), representing a novel group D related cluster, was less prevalent. Seven isolates (8.0%) were assigned to phylogenetic group E and one isolate (1.1%) belonged to group G. Phylogroups B2, frequently harboring more pathogenic *E. coli*, and group C were not detected in this study.

Multi Locus Sequence Typing following the Achtmann scheme assigned 85 of the isolates to 35 different sequence types (STs), while the allele profile of two strains (BfR-23-MO00238, BfR-23-MO00269) does not match to a designated ST of the pubMLST database. There are 20 STs, which are represented by more than one isolate, with ST1011 being the most prevalent (n = 8) detected in isolates from the chicken companies A, B and C. The other ST types were ST10 (n = 6), ST1882 (n = 6), ST58 (n = 5), ST1485 (n = 5), ST93 (n = 4), ST162 (n = 4), ST38 (n = 4), ST155 (n = 3), ST189 (n = 3), ST212 (n = 3), ST746 (n = 3), ST4274 (n = 2), ST624 (n = 2), ST101 (n = 2), ST219 (n = 2), ST770 (n = 2), ST6027 (n = 2), ST648 (n = 2), and ST744 (n = 2). The remaining 15 isolates represented individual STs (ST57, ST69, ST117, ST156, ST295, ST354, ST457, ST665, ST752, ST1431, ST1723, ST3941, ST4373, ST7941 and ST10343).

### Phylogenetic SNP analysis

**Fig 2:**
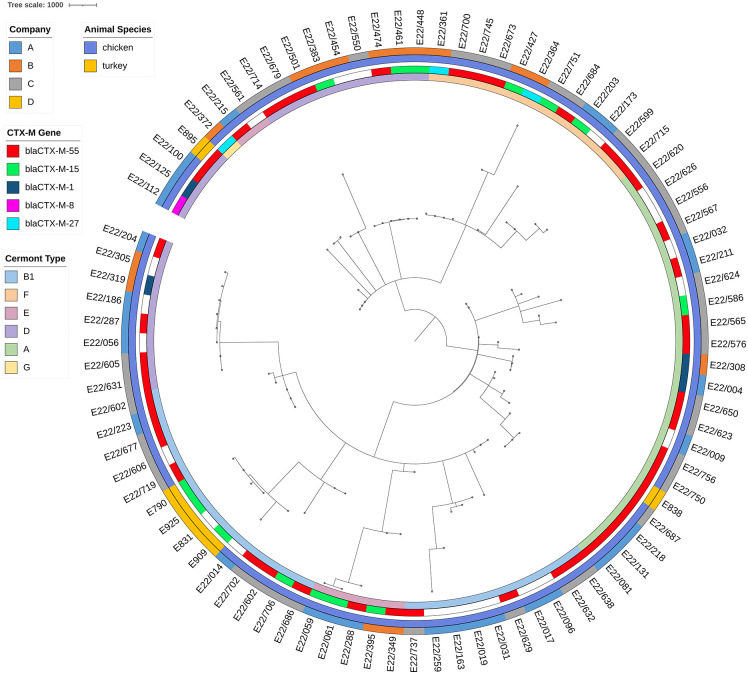
cgMLST based phylogenetic tree of the studied isolates of ESBL-producing *E. coli* from chicken and turkey companies, calculated based on cgMLST (chewiesnake) and visualized with iTol v7. The isolate IDs are shown around the circular illustration. From the outside to the inside the four circles show the poultry companies (A, B, C and D), the animal species of the sample (chicken and turkey), the detected CTX-M genes and the Clermont types (phylogenetic group) they belong to, differentiated by color codes as given in the legend.

A phylogenetic tree was inferred from the cgMLST results of all 87 *E. coli* strains (Fig 2). A distance threshold of 10 revealed 69 distinct clusters, highlighting the high genetic diversity within the tested *E. coli* population. The largest cluster consisted of four *E. coli* isolates from four different farms, all belonging to company B. Beyond these clonal clusters, isolates originating from different regions and farms were interspersed throughout the phylogenetic tree, suggesting that genetically diverse ESBL-producing *E. coli* strains are widely distributed across poultry production areas in western Türkiye.

### *Escherichia coli* genomes exhibit a broad set of plasmid types of which especially IncHI2A and IncFIB carry ESBL and AmpC determinants

PlasmidFinder was used to predict at least one plasmid replicon type in each of the ESBL-producing *E. coli* (S1 Table). The most frequent replicon sequences were: IncFIB (AP001918) (n = 60), Col (MG828) (n = 42), ColRNAI (n = 40), IncFIC (FII) (n = 39), Col440I (n = 22), IncI1 (Alpha) (n = 19), pKPC-CAV1321 (n = 18), p0111 (n = 18), IncHI2A (n = 18), IncFIA (n = 16), Col156 (n = 14) and IncFIB(pHCM2) (n = 10). The other less frequently found replicon types are IncX1 (n = 7), ColpVC (n = 6), IncB/O/K/Z (n = 6), IncX4 (n = 5), IncFII (pCoo) (n = 5), Col8282 (n = 5), IncQ1 (n = 4), IncY (n = 4), IncI2 (Delta) (n = 4), IncHI2 (n = 4), IncFII (29) (n = 3), pUTI89 (n = 3), Col(MP18) (n = 2), IncX3 (n = 1), IncN (n = 1), BS512 (n = 1), ColE10 (n = 1), IncFIB (pB171) (n = 1), IncFII (pRSB107) (n = 1), IncFII (pSE11) (n = 1) and IncFIA (HI1) (n = 1).

In 26 of the isolates the contig harboring the ESBL or AmpC gene also harbored plasmid markers. In five isolates *bla*_CTX-M-15_ was associated with IncFIB type plasmid, in one with an p0111. Two isolates harbored *bla*_CTX-M-1_ genes associated with a IncI1 plasmid, another isolate a *bla*_CTX-M-55_ with an IncFIC plasmid. *bla*_CMY-2_ was frequently associated with plasmids. In nine isolates it was found together with IncHI2A, in eight isolates with an IncI1 and in two isolates with and IncB plasmids.

### Virulence genes

In this study, numerous virulence-associated genes (VAGs) have been detected in the genomes of ESBL-producing *E. coli*. Using the WGS data, VAGs were selected from public databases included in the *E. coli* functional genotyping. We identified 58–124 VAGs in all ESBL-producing *E. coli* strains using WGS. In-silico, the genomes were screened for the presence of adherence, antimicrobial activity/competitive advantage, effector delivery system, immune modulation, invasion, nutritional/metabolic factor and regulation groups of virulence. In this collection, the most uniformly conserved virulence factors, detected in 100% of the strains, were *ompA*, *ibeC*, *entA-E*, *fepB*, *phoP*, *rpoS*, *rcsB*, *fur*, and *tssA/tssM*. These highly conserved genes are mainly related to adhesion, iron acquisition, stress response, and secretion systems, indicating a strong adaptive potential of the isolates.

## Discussion

Since 2014, member states of the European Union (EU) have been using *E. coli* as a common commensal indicator bacterium for monitoring AMR in farm animals [7]. To the best of our knowledge, there is no comparable regulatory framework implemented in Türkiye. Therefore, this preliminary study was carried out to establish a baseline data for the future investigations.

Studies including *E. coli* isolates from poultry and other farm animals, both commensal and pathogenic, have demonstrated resistance to multiple antibiotics, including TET, NAL, SMX, TYL, PEN and AMP [14,27]. Although there are a number of existing local reports, in which isolates were collected without uniformed isolation criteria, and such inconsistency makes the comparison difficult. Despite these limitations, the resistance patterns observed in the present study are largely comparable to those reported from poultry production system in Europe and other regions [10,12,28]. Our findings show a clear host-associated divergence in resistance patterns, as CIP, AMC, FFC, CHL and SXT resistance was found to be significantly higher among *E. coli* isolates from chickens, when compared to those obtained from turkey. On the other hand, doxycycline resistance was remarkably higher among *E. coli* isolates from turkey farm than those obtained from chicken farms. This difference could be attributed to comparatively limited antibiotic usage in turkey production, nonetheless, only farms of one turkey producer was included, representing a limitation for this study.

Our study found a very high rate of resistance to TYL, AMP, and T among commensal *E. coli* from poultry samples in Türkiye. These findings closely mirror results from both national and international poultry studies, in which resistance to older and commonly used antimicrobials remains widespread [10,14,28,29]. High resistance rates to AMP, SMX, CIP, and NAL have been consistently reported in both national studies, European Union surveillance reports, and large scale meta analyses across Europe and globally [9,12,29]. On the other hand, lower AMP resistance levels among *E. coli* from chickens and fattening turkeys in the European report was also reported to be 46.6% and 55.8%, respectively [9]. Additionally, significant resistance to CIP (48.4%), a critically important antimicrobial, was observed in the current study, reflecting concerns about widespread resistance to quinolone antibiotics in poultry [30]. In contrast, a recent study from Australia reported a markedly low CIP resistance rate (3.3%) among poultry-associated *E. coli*, which has been attributed to the long-standing restriction of quinolone use in poultry production and as well as resitrictions on the importation of live poultry and fresh meat [11]. This comparison highlights the strong association between national antimicrobial usage policies and the emergence of fluoroquinolone resistance in poultry-associated *E. coli*.

In our study, resistance to cefotaxime and/or ceftazidime differed by poultry company and species, remaining low in fattening turkeys (4.0%) and chickens from companies A and B (7.6%−11.8%), while reaching higher levels in chickens from company C (20.4%). Notably, a recent large-scale Australian study analyzing *E. coli* isolates from approximately 2,950 meat chickens reported no resistance to FOT or TAZ [11]. Differences likely reflect variations in antimicrobial usage practices among companies. In addition, 11.4% of the *E. coli* isolates recovered from poultry caecal samples (98 chickens and seven fattening turkeys) were identified as ESBL-producing strains (n = 105/918). This proportion reflects the frequency among recovered isolates, not the prevalence at sample level, as selective screening for ESBL-producing *E. coli* was not performed on all caecal samples. According to EFSA/ECDC [31], the proportion of ESBL-producing *E. coli* strains in chickens across EU member states ranges from 0.6% to 7.1%, while values in fattening turkey range between 0.6% and 6.3%. Similarly low strain-based frequencies have been reported in Asian countries, including Malaysia (5.5%) [32] and South Korea (7.0%) [33]. However, higher strain-based frequencies have also been reported in Hungary (34.2%) [34] and Italy (43.6%) [35], as well as in African settings, such as Ghana (29.0%) [36]. Similarly, ESBL producing *E. coli* frequencies in turkeys vary internationally, ranging from 2.2% in Portugal [37] to 5.0% in Canada [38] and 6.8% in Egypt [39].

WGS analysis demonstrated a broad genetic diversity of β-lactamase determinants within the *E. coli* population, including *bla*_CTX-M_, *bla*_TEM_, *bla*_OXA_, and *bla*_CMY-2_ variants, which is consistent with previous reports [13]. In our study, the predominant ESBL genes were *bla*_CTX-M-55_ and *bla*_CTX-M-15_, while *bla*_CMY-2_ and *bla*_DHA-1_ were the main AmpC genes identified. While comparative data from other countries show that *bla*_CTX-M-1_ can occur at much higher rates, such as in poultry feces and farm environments in Germany (41%) [40] and chicken isolates in Canada (91%) [41], the proportion of *bla*_CTX-M-1_ detected in our study was notably lower. Several earlier studies have also reported that other β-lactamase genes, including *bla*_SHV_ and *bla*_CMY-2_, may be more prevalent than *bla*_CTX-M_ variants [42–44]. Such variability has been attributed to factors such as antimicrobial usage patterns, environmental and geographical characteristics, and plasmid mobility [45].

A notable trend observed globally is the shift from *bla*_CTX-M-15_ to *bla*_CTX-M-55_ in poultry-associated *E. coli*. While *bla*_CTX-M-15_ had previously been dominant in countries such as Germany and various African countries [36,46], Türkiye was among the first to report a rapid increase in *bla*_CTX-M-55_ prevalence [16,17]. Similar transitions have been documented in other regions, including China [47–49]. Consistent with these observations, Dugget et al. [49] reported that while *bla*_CTX-M-1_ was the most common ESBL gene in poultry isolates from 2016−2018, by 2020 the dominant variant had shifted to *bla*_CTX-M-55_. Additionally, ESBL-producing *E. coli* from chickens have been shown to transfer β-lactam resistance via extracellular vesicle (EV)-mediated horizontal gene transfer, providing a plausible mechanism for the rapid dissemination of these genes. Recent evidence also suggests that ESBL-producing *E. coli* may disseminate *bla*_CTX-M-55_ through EV-mediated horizontal gene transfer. Xu et al. [50] reported that EV-mediated transfer of *bla*_CTX-M-55_ is not random; rather, it appears to be selective and may occur more efficiently between closely related bacterial species. Additionally, the detection of CTX-M-55 across multiple plasmid backbones in livestock-associated *E. coli* raises concern that the gene is becoming increasingly mobile, thereby enhancing its capacity for rapid spread within and between bacterial populations. Given this mobility, routine genetic monitoring of CTX-M variants is essential for detecting emerging mutations and tracking the dissemination pathways of ESBL genes. Strengthening surveillance systems and developing targeted control strategies are therefore critical to limit the further expansion of *bla*_CTX-M-55_ within both animal and human reservoirs.

Phenotypic resistance patterns showed good agreement with resistance determinants detected by WGS using ResFinder. Among these findings, colistin resistance was detected in a single isolate, which was confirmed by the presence of the plasmid-mediated *mcr-*1 gene. This observation is in line with previous studies reporting a low prevalence of *mcr*-1 among retail poultry meat in Türkiye [18,51]. Although *mcr*-1 remains infrequently detected, its occurrence in the poultry production chain highlights the importance of continued surveillance due to its potential for horizontal dissemination.

In our study, ESBL-producing *E. coli* strains exhibited a diverse range of phylogroups, with B1 being the most common (27.6%), followed by A (25.3%), D (24.1%), and F (13.8%). Also, phylogroup E and G were detected in low proportions. Notably, one isolate was identified as belonging to the G phylogroup, an intermediary between F and B2. These findings align with previous studies, which indicate that phylogroups A and B1 are typically commensal, while B2, D and E are often pathogenic; the presence of D phylogroup isolates suggests potential for commensal strains to become pathogenic under certain conditions [13].

The high number of STs, together with the wide SNP variation observed in the isolates, suggests multiple contamination sources rather than clonal expansion, a pattern similarly described in the poultry production chain. Despite this diversity, ST1011 and ST10 were the most frequent STs. Notably, six of the top 20 ExPEC-associated lineages were detected (ST10, ST38, ST58, ST69, ST117, ST354), all of which are well-recognized contributors to both human and animal infections [52]. ST10 is widely reported as a dominant MDR lineage in animals [53]. Notably, a single ST69 isolate carried *bla*_CMY-2_, a lineage previously described as globally disseminated in poultry [54].

In this study, ESBL-producing *E. coli* isolates carried a broad set of VAGs, including adhesion factors (e.g., *fim*, *papC* and *sfa*), enterotoxin genes (*est*), the intimin gene (*eae*), and iron acquisition systems such as *iutA*, *fyuA*, and *iucC*. The presence of these genes aligns with previously reported virulence profiles of avian ExPEC-related lineages [13]. While the detection of these VAGs indicates that some isolates possess traits associated with extra-intestinal pathogenicity, our data do not allow conclusions regarding zoonotic transmission. Instead, these findings highlight the need for continuous surveillance to monitor the co-occurrence of virulence and resistance determinants in poultry-associated *E. coli* populations.

Plasmids play a crucial role in the worldwide spread of ESBL genes [12]. Previous studies have identified variable plasmid replicon types including IncFIB, Col(MG828), IncFII, IncFIC(FII) and IncX1 [33,55]. In the current study, IncFIB, Col(MG828) and ColRNAI were the most frequent replicon types encountered in ESBL producing *E. coli*, followed by IncFIC(FII), Col440I and IncI1 (Alpha). In South Korea, *bla*_CTX-M-55_ positive *E. coli* isolates have also been reported to carry IncF plasmids in combination with other replicon types such as FIB, I1-Ig, K, N, and/or FIA [33]. Overall, these findings indicate that IncF and Col-type plasmids constitute common and well-adapted genetic backbones in poultry-associated *E. coli* populations across different regions, facilitating the maintenance and dissemination of ESBL determinants within poultry production systems.

## Conclusion

In this study, high resistance to TYL, AMP, T, TET, FFL, and SXT was found among commensal *E. coli* isolates from poultry, with marked differences in resistance prevalence between poultry companies. WGS analysis provided detailed insights into the genetic characteristics of these strains, revealing diverse MLST profiles, with ST1011 identified as the most frequent sequence type. Among ESBL producing isolates, the *bla*_CTX-M-55_ gene emerged as the most common ESBL determinant, frequently associated with transferable plasmid replicon types, indicating a high potential for dissemination within the poultry production chain. Although the study was limited by the lack of detailed farm-level antimicrobial usage data, these findings provide valuable baseline information for Türkiye and highlights the need for continuous, harmonized AMR surveillance and targeted control strategies in the poultry sector.

## Supporting information

S1 Table WGS data of ESBL-producing *Escherichia coli* isolates. (XLSX)
